# Supplementary material for: Cytokinin Regulates Energy Utilization in Botrytis cinerea
Source: Microbiol Spectr. 2022 Jul 27;10(4):e00280-22. doi: 10.1128/spectrum.00280-22 (PMC9430538; doi:10.1128/spectrum.00280-22)
Supplement: Supplemental file 1 — Supplemental material. Download spectrum.00280-22-s0001.pdf, PDF file, 0.9 MB [file spectrum.00280-22-s0001.pdf]

Supplemental Materials

Fig. S1. CK-mediated growth inhibition in PDA depends on media strength.

Fig. S2. CK-mediated growth inhibition in PDB depends on media strength**.**

Fig. S3: CK-mediated growth effects depend on glucose availability- light experiments**.**

Fig. S4: Acidification levels in different synthetic media with and without CK.

Fig. S5. CK-mediated growth inhibition levels depend on growth rate**.**

Fig. S6. CK rescues glycolysis inhibition and partially rescues ATP synthesis inhibition**.**

Fig. S7. CK promotes glucose utilization in PDB.

Fig. S8. Plant endogenous CK alters *B. cinerea* cytosolic redox state during infection-additional genotypes.

Fig. S9. Plant endogenous CK alters *B. cinerea* redox state during infection- plate assay.

Fig. S10. *B. cinerea* transformed roGFP lines display virulence behaviour similar to that of the background line.

Fig. S11. CK alters *B. cinerea* redox state- changes in the transcriptome.

Table S1. Oligonucleotides used for generating and validating *Botrytis cinerea* transgenic strains.

Table S2. Primers used in RT-qPCR.

Data S1. Transcriptomic effect of CK on *B. cinerea* metabolic pathways.

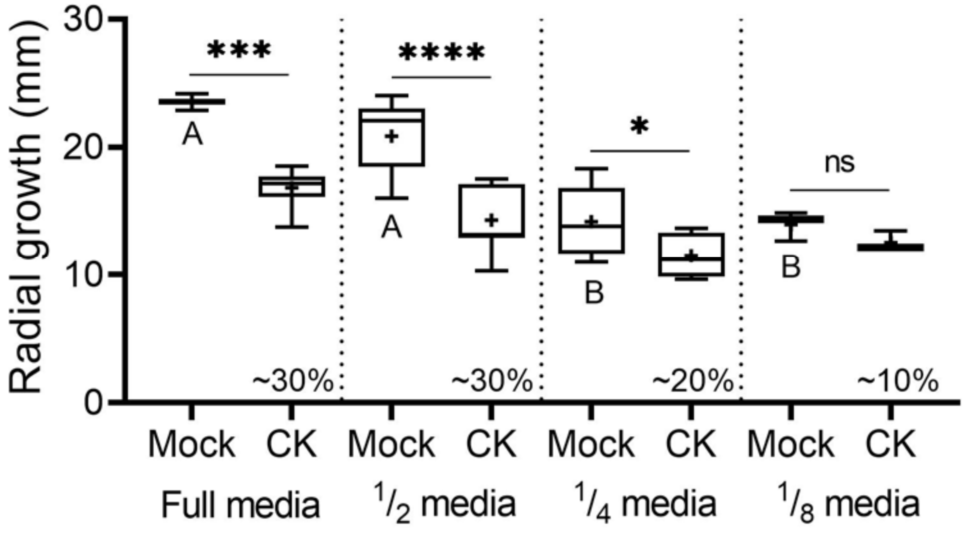

Fig. S1: CK-mediated growth inhibition in PDA depends on media strength.

B. cinerea mycelia were grown on PDA plates without (Mock) or with the addition of the CK 6-BAP (6-Benzylaminopurine, 100 µM) and incubated at 22 ± 2 °C in the dark. Mycelial area was measured after 5 days. Boxplots are shown with minimum to maximum values, inner quartile ranges (box), median (line in box), mean ("+" sign in box), and outer quartile ranges (whiskers), N=6. Results were analyzed for statistical significance using a one-way ANOVA with a Bonferroni post-hoc test, or a two-tailed t-test with Welch's correction. Asterisks indicate statistically significant differences between the Mock and CK samples within the same media, ****p<0.0001; *p<0.05; ns=non-significant. Upper case letters indicate statistically significant differences in the growth of Mock samples in different media, p<0.0035; lower case letters indicate statistically significant differences in the growth of CK-treated samples in different media, p<0.05.

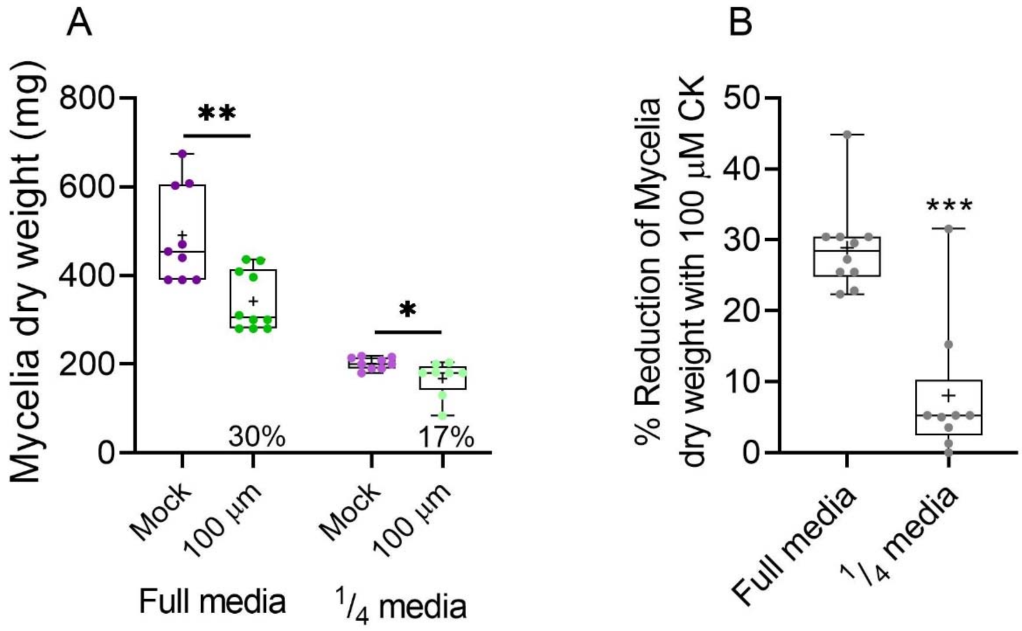

Fig S2: CK-mediated growth inhibition in PDB depends on media strength.

B. cinerea spores (10^6/ mL dissolved in sterile water) were grown in stationary liquid full or 1/4 PDB media, at 22 ± 2 °C in the dark, without (Mock) or with the addition of the CK 6-BAP (6-Benzylaminopurine, 100 µM). After 3 days, the samples were dried and weighed. **(A)** Mycelia weight. The percent of reduction with CK is indicated. **(B)** Percent of reduction in growth is compared between full and 1/4 media. Boxplots are shown with minimum to maximum values, inner quartile ranges (box), median (line in box), mean ("+" sign in box), and outer quartile ranges (whiskers), N=9. Results were analyzed for statistical significance using a one-way ANOVA with a Bonferroni post-hoc test, or a two-tailed t-test with Welch's correction. Asterisks indicate statistically significant differences between the Mock and CK samples within the same media (A), or statistically significant differences between the effect of CK in full and 1/4 media (B). ***p<0.001; **p<0.01; *p<0.05.

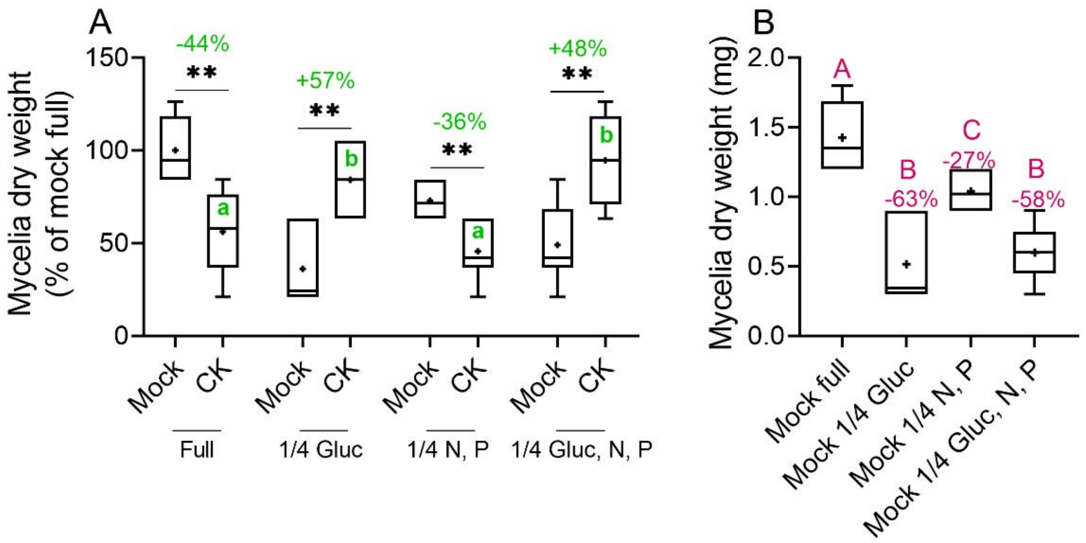

Fig. S3: CK-mediated growth effects depend on glucose availability- light experiments.

B. cinerea spores were inoculated in 4 types of defined media: Full (20 g/L glucose, and 4 g/L each of K_2HPO_4, KH_2PO_4, and $(NH_4)_2SO_4$), 1/4 glucose (5 g/L glucose, and 4 g/L each of K_2HPO_4, KH_2PO_4, and $(NH_4)_2SO_4$), 1/4 Nitrogen and phosphate (20 g/L glucose, and 1 g/L each of K_2HPO_4, KH_2PO_4, and $(NH_4)_2SO_4$), and 1/4 glucose, nitrogen, and phosphate (5 g/L glucose, and 1 g/L each of K_2HPO_4, KH_2PO_4, and $(NH_4)_2SO_4$). Samples were prepared without (Mock) or with the addition of the CK 6-BAP (6-Benzylaminopurine, 100 µM). Germinated spores were grown with shaking (150 rpm) at 22 ± 2 °C in constant LED light (maximum light intensity of 450 µmol m^{-2} s^{-1}), for 3 days, after which the fungal matter was dried and weighed. Boxplots are shown with minimum to maximum values, inner quartile ranges (box), median (line in box), mean ("+" sign in box) and outer quartile ranges (whiskers). Results were analyzed for statistical significance using one-way ANOVA with Tukey's post-hoc test, or a two-tailed t-test with Welch's correction.

A. Asterisks indicate statistically significant differences between the Mock and CK samples within the same media, $**p>0.01$, N=6. Different lower case letters indicate statistically significant differences in the growth of CK-treated samples in different media, $p<0.045$. Percentages of the effect of CK on fungal growth in the different media are indicated above the asterisks.

B. Comparison of Mock samples in the different media. Different upper case letters indicate statistically significant differences between mock samples grown in the indicated media. The percentage of growth inhibition in the different media as compared with Full medium is indicated above the upper whisker for each medium. N=6, $p<0.033$.

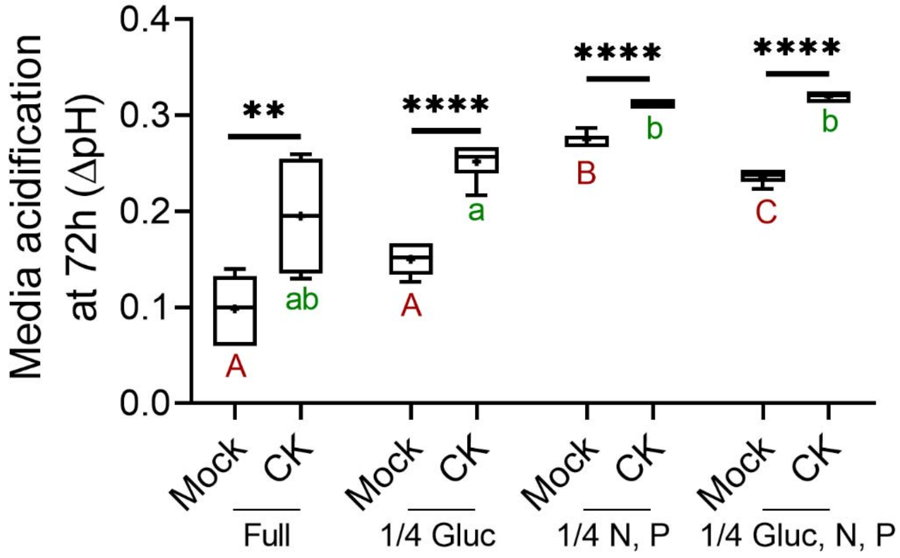

Fig. S4: Acidification levels in different synthetic media with and without CK.

B. cinerea spores were inoculated in 4 types of defined media: Full (20 g/L glucose, and 4 g/L each of K_2HPO_4, KH_2PO_4, and $(NH_4)_2SO_4$), 1/4 glucose (5 g/L glucose, and 4 g/L each of K_2HPO_4, KH_2PO_4, and $(NH_4)_2SO_4$), 1/4 Nitrogen and phosphate (20 g/L glucose, and 1 g/L each of K_2HPO_4, KH_2PO_4, and $(NH_4)_2SO_4$), and 1/4 glucose, nitrogen, and phosphate (5 g/L glucose, and 1 g/L each of K_2HPO_4, KH_2PO_4, and $(NH_4)_2SO_4$). Samples were prepared without (Mock) or with the addition of the CK 6-BAP (6-Benzylaminopurine, 100 µM). Germinated spores were grown with shaking (150 rpm) at 22 ± 2 °C in the dark, for 3 days, after which the fungal matter was centrifuged, and pH measured in the supernatant. Boxplots are shown with minimum to maximum values, inner quartile ranges (box), median (line in box), mean ("+" sign in box) and outer quartile ranges (whiskers). Results were analyzed for statistical significance using Welch's ANOVA with Dunnett's post-hoc test.

Asterisks indicate statistically significant differences between the Mock and CK samples within the same media, ****p<0.0001; **p>0.01, N=6. Different uppercase letters indicate statistically significant differences in media acidification of Mock samples in different media, p<0.0012. Different lower case letters indicate statistically significant differences in media acidification of CK-treated samples in different media, p<0.0032.

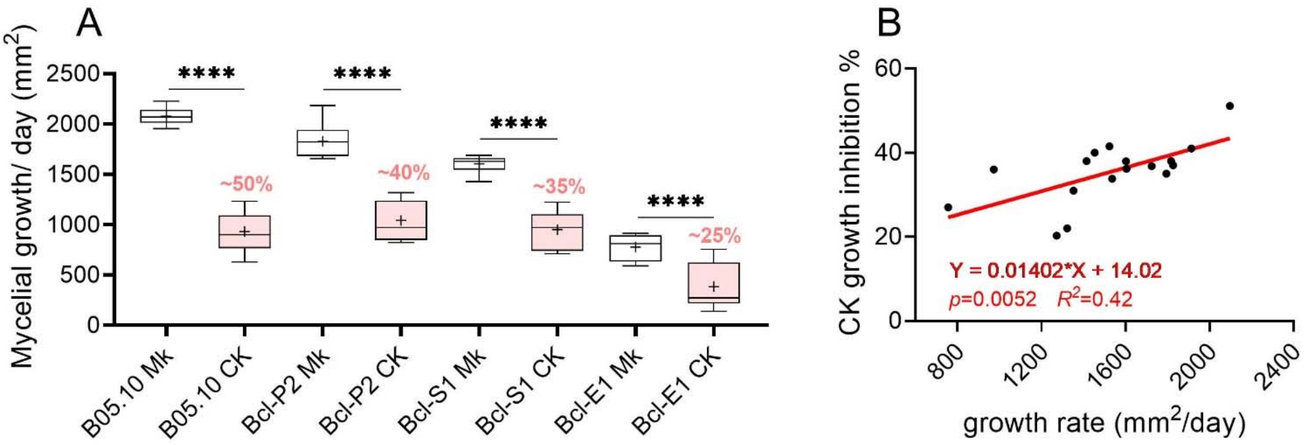

Fig. S5: CK-mediated growth inhibition levels depend on growth rate.

B. cinerea mycelia from different isolates were grown on PDA plates without (Mock) or with the addition of the CK 6-BAP (6-Benzylaminopurine, 100 µM) and incubated at 22 ± 2 °C in the dark. Mycelial area was measured after 5 days. Experiment was repeated 3 independent times, N=9.

A growth rate per day of several isolates with or without CK. Boxplots are shown with minimum to maximum values, inner quartile ranges (box), median (line in box), mean ("+" sign in box), and outer quartile ranges (whiskers). Results were analyzed for statistical significance using a one-way ANOVA with a Bonferroni post-hoc test. Asterisks indicate statistically significant differences between the Mock and CK samples of each isolate, ****p<0.0001. The average % of CK mediated inhibition is indicated above the CK bar for each isolate.

B Regression analysis of the relationship between growth rate and CK-mediated inhibition in 17 different *B. cinerea* isolates from tomato, pepper, eggplant, cucumber, grapevine, and strawberry. Simple linear regression among samples was found significantly different from zero, p=0.0052, Pearson correlation coefficient- R^2=0.042.

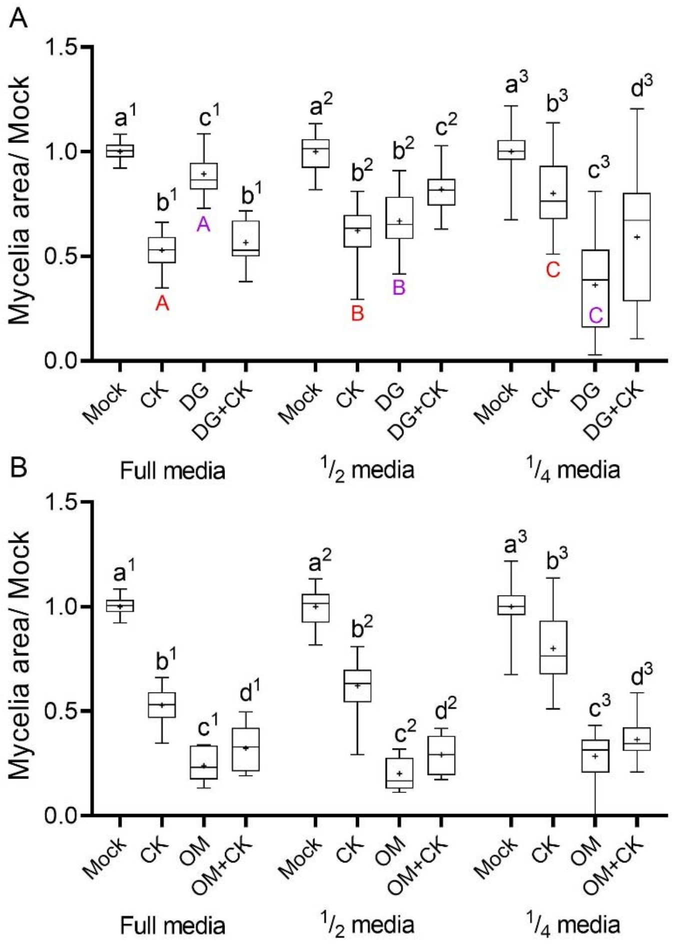

Fig. S6: CK rescues glycolysis inhibition and partially rescues ATP synthesis inhibition.

B. cinerea mycelia were grown on PDA plates without (Mock) or with the addition of the CK 6-BAP (6-Benzylaminopurine, 100 µM), the competitive glucose inhibitor 2-DG (2-deoxyglucose, 2.5 mM) (**A**), or the ATP synthesis inhibitor OM (oligomycin, 0.1 µg/ml) (**B**) and incubated at 22 ± 2 °C in the dark. Mycelia area was measured after 5 days. Boxplots are shown with minimum to maximum values, inner quartile ranges (box), median (line in box), mean ("+" sign in box), and outer quartile ranges (whiskers), N=10.

A: 2-deoxyglucose (DG). Results were analyzed for statistical significance using a one-way ANOVA with a Tukey post-hoc test. Lower case letters indicate statistically significant differences between samples, with number tags indicating the group that was comparatively analyzed, $p<0.025$. Upper case letters within the top of CK bars indicate statistically significant differences in the level off CK-mediated growth inhibition, $p<0.018$. Upper case letters within the bottom of DG bars indicate statistically significant differences in the level off DG-mediated growth inhibition, $p<0.011$.

B: Oligomycin (OM). Results were analyzed for statistical significance using a one-way ANOVA with a Tukey post-hoc test, or a two-tailed t-test with Welch's correction. Letters indicate statistically significant differences between samples, with tags indicating the group that was comparatively analyzed, $p<0.038$.

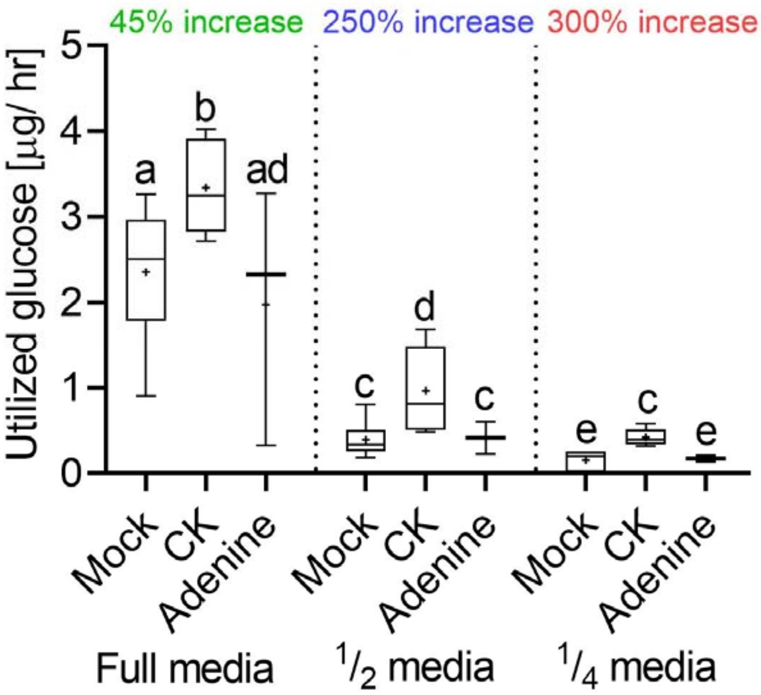

Fig. S7: CK promotes glucose utilization in PDB.

B. cinerea spores (10^6/ mL dissolved in sterile water) were grown in PDB with 150 rpm shaking, at 22 ± 2 °C in the dark, without (Mock) or with the addition of the CK 6-BAP (6-Benzylaminopurine, 100 µM), or the structural control Adenine, 100 µM. The amount of glucose in the media was examined after 48 h, and subtracted from the amount of glucose present in media without fungi that underwent similar treatment. The approximate percent of increase in glucose uptake in the presence of CK is indicated above the bars for each media concentration. Boxplots are shown with minimum to maximum values, inner quartile ranges (box), median (line in box), mean ("+" sign in box), and outer quartile ranges (whiskers), N=6. Results were analyzed for statistical significance using two-tailed t-test with Welch's correction. Letters indicate statistically significant differences between samples, $p<0.04$.

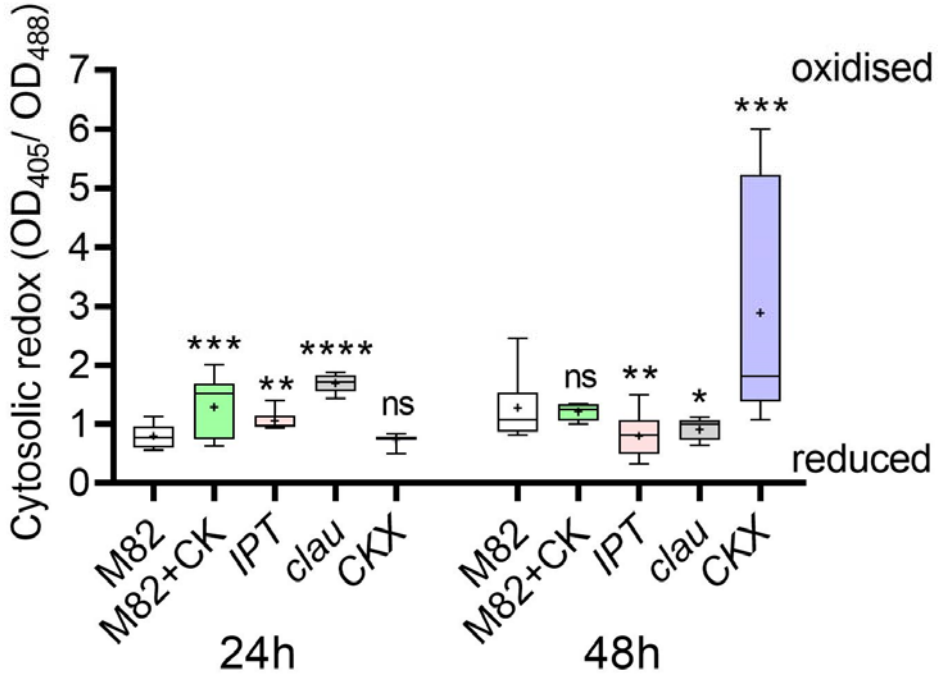

Fig S8: Plant endogenous CK alters *B. cinerea* cytosolic redox state during infection- additional genotypes.

The redox state of *B. cinerea* when infecting leaves of different CK-content tomato genotypes was assessed using roGFP transformed *B. cinerea*. Spores (10^6/ mL in glucose and K_2HPO_4) of *B. cinerea* expressing GRX-roGFP, for assessing cytosolic redox state, were used to infect the background M82 wild-type line, M82 treated with exogenous CK (100 µM 6-BAP) by spraying, 24 h before inoculation ("M82+CK"), the high-CK *pBLS>>IPT7* overexpressing line ("*IPT*"), the CK hypersensitive mutant *clausa* ("*clau*"), and the low-CK *pFIL>>CKX3* overexpressing line ("*CKX*").

B. cinerea fluorescence was captured using a confocal laser scanning microscope at 24 h and 48 h, with excitation at 405 nm for the oxidized state and 488 nm for the reduced state of roGFP2. The emission was detected using a 505-530 nm bandpass filter. The redox ratio of the fungus was calculated as Em405/Em488 using ImageJ, from at least 8 images per time point, per treatment. Boxplots are shown with minimum to maximum values, inner quartile ranges (box), median (line in box), mean ("+" sign in box), and outer quartile ranges (whiskers), N=8. Asterisks indicate significant differences from the redox state of *B. cinerea* infecting the M82 background line, assessed using a one-way ANOVA with a Dunnett post hoc test. **p*<0.05, ***p*<0.01, ****p*<0.001, *****p*<0.0001.

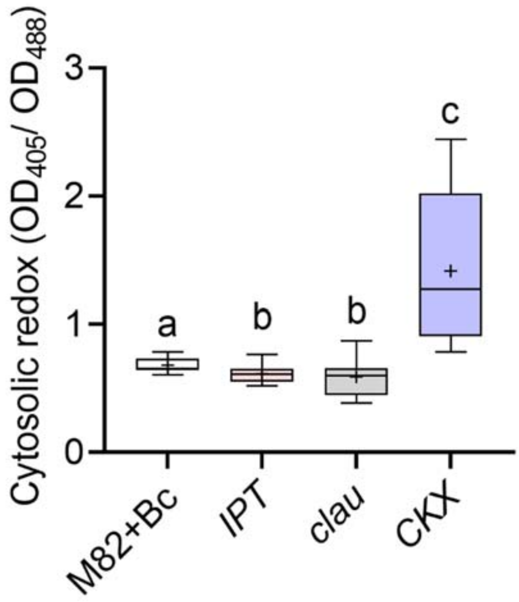

Fig S9: Plant endogenous CK alters *B. cinerea* cytosolic **redox state during infection- plate assay.**

The redox state of *B. cinerea* when infecting leaves of different CK-content tomato genotypes was assessed using roGFP transformed *B. cinerea*. Spores (10^6/ mL in glucose and K_2HPO_4) of *B. cinerea* strains expressing GRX-roGFP, for assessing cytosolic redox state, were used to infect leaf discs of the background M82 wild-type line, the high-CK *pBLS>>IPT7* overexpressing line ("IPT"), and the low-CK *pFIL>>CKX3* overexpressing line ("CKX"). Infected leaf discs were incubated in a 96-well plate for 48 h at 18 °C. Fluorescence was measured using a fluorimeter, with excitation at 405 ± 5 nm for the oxidized state and 488 ± 5 nm for the reduced state of roGFP2. The emission was detected at 510 ± 5 nm. Samples were normalized to the readings of each genotype without the fungus. The redox ratio of the fungus was calculated as Em405/Em488 of Relative fluorescence units (RFU). Boxplots are shown with minimum to maximum values, inner quartile ranges (box), median (line in box), mean ("+" sign in box), and outer quartile ranges (whiskers), N=22. Differences between samples were assessed using a one-way ANOVA with a Dunnett post hoc test. Letters indicate statistically significant differences between samples, $p<0.0038$.

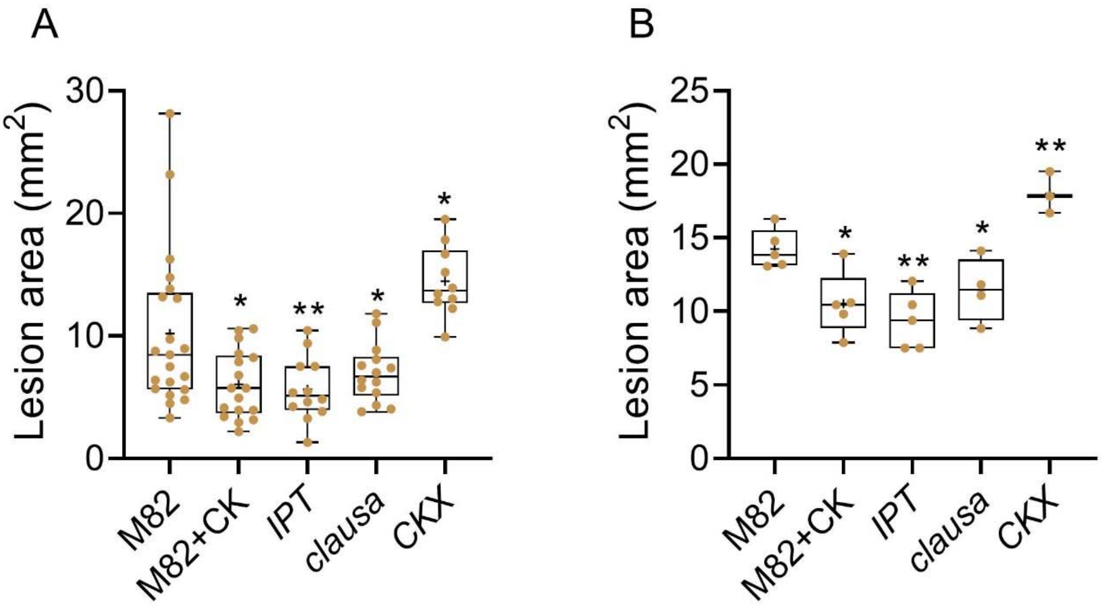

Fig S10: ***B. cinerea*** **transformed roGFP lines display virulence behavior similar to that of the background line.**

Spores (10^6/ mL in glucose and K_2HPO_4) of *B. cinerea* strains expressing GRX-roGFP, for assessing cytosolic redox state (**A**), and mito-roGFP, for assessing mitochondrial redox state (**B**), were used to infect leaves of the background M82 wild-type line, M82 treated with exogenous CK (100 µM 6-BAP) by spraying, 24 h before inoculation ("M82+CK"), the high-CK *pBLS>>IPT7* overexpressing line ("IPT"), the CK hypersensitive mutant *clausa* ("clau"), and the low-CK *pFIL>>CKX3* overexpressing line ("CKX"). Lesion area was measured 5 days post inoculation using ImageJ. Boxplots are shown with minimum to maximum values, inner quartile ranges (box), median (line in box), mean ("+" sign in box), and outer quartile ranges (whiskers), (A) N>10, (B) N>3. Asterisks indicate significant differences from the disease levels in the M82 background line, assessed using a one-way ANOVA with a Dunnett post hoc test (A), or a two-tailed t-test with Welch's correction (B); *p<0.05, **p<0.01, ***p<0.001, ****p<0.0001.

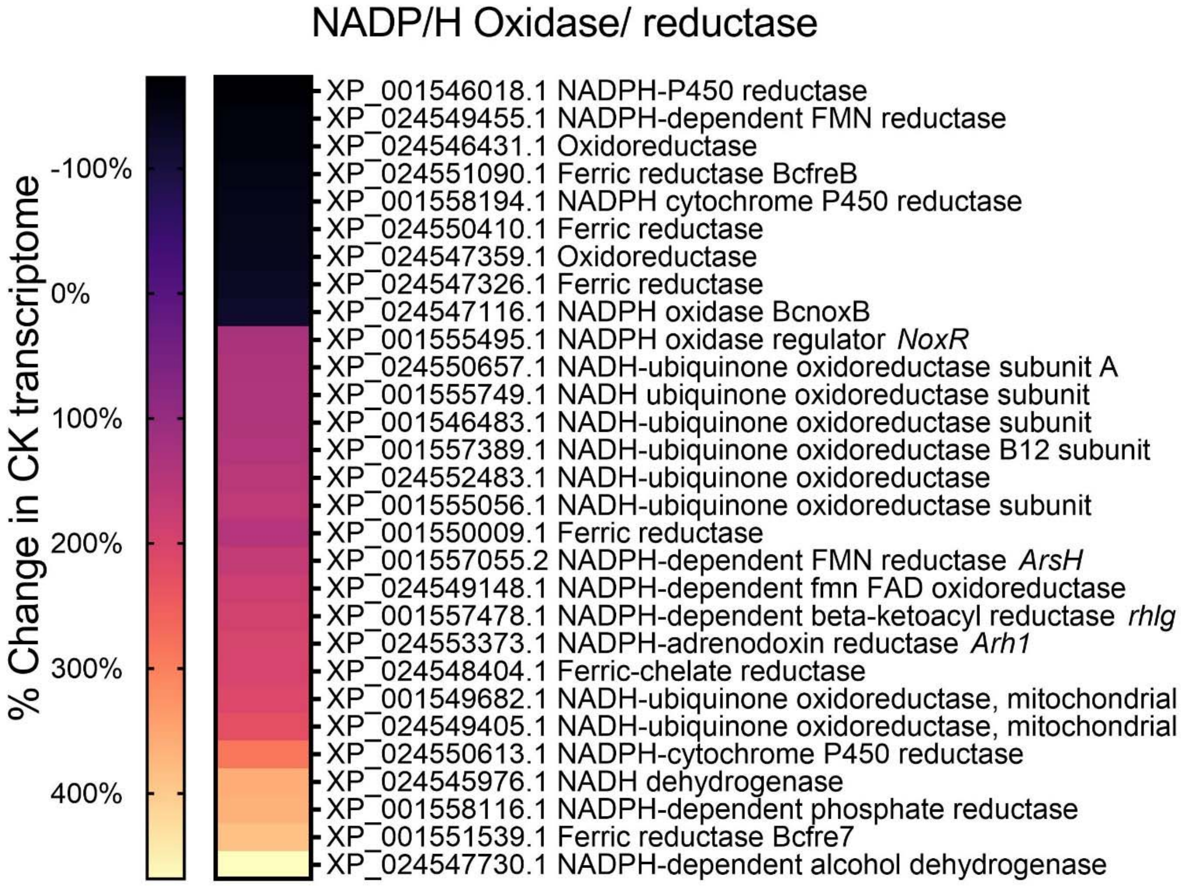

Fig S11: CK alters *B. cinerea* redox state- changes in the transcriptome.

Illumina Hiseq NGS was conducted on *B. cinerea* Mock treated or CK treated samples, 3 biological repeats each. Gene expression values were computed as FPKM, and differential expression analysis was completed using the DESeq2 R package. Genes with an adjusted *p*-value of no more than 0.05 and $\log_2$FC (Fold Change) greater than 1 or lesser than -1 were considered differentially expressed. The full transcriptome data was previously published, and is available (NCBI bioproject PRJNA718329). Changes in NADP/H oxido-reductases in the transcriptome of CK treated *B. cinerea* are plotted on a heatmap.

Table S1. Oligonucleotides used for generating and validating *Botrytis cinerea* transgenic strains.

Primer	Sequence (5’-3’)
GA 34F	CGGGTGAATGGGATTCATTG
GA 34R	GCCCGCATTGGATTAATAATTG
GA 44F	GCCACAGACTCCGCCAGATTCTAATG
GA 44R	CAACCATTTCACGCTGCGACCACC
GA 31F	GCAACTAGTGATATTGAAGG
GA 31R	CATCTACTCTATTCCTTTGC
GA 41F	AGGGTTTTCCCAGTCACGACG
GA 41R	GCGGATAACAATTTCACACAG
GA 42F	GCGGGGTATGGCAGCATGAGTG
GA 42R	CTTATAGCAAGCGCGATGTGTATC

Table S2. Primers used in RT-qPCR.

Gene	Sequence (5’-3’)	Primer Efficiency
Glyceraldehyde-3-phosphate dehydrogenase (*GAPDH*)	F- CGCCGTCATCCCTTTT<br>R- TTGACCGTGGGTGGAA	0.97
Pyruvate dehydrogenase (*PYDH*)	F- CCAAGGAATACGCCAACG<br>R- CCAGCGATTGGATCTTGG	1.03
Aldehyde dehydrogenase (*ADH*)	F- TCCATCGCCAAGTTCTCC<br>R- CCAATGCCGGATTCTTTG	0.97
Alcohol dehydrogenase (*ALDH*)	F- GAGGGCAAGATTGCTGGA<br>R- CGATGCACGCTCAAACAC	1.01
Purine nucleoside permease (*PUP1*)	F-TGAGTTCGGTGGTGAGCA<br>R-TGACCCGACACTGAAGCA	0.98
Purine-cytosine permease (*PUP2*)	F-CCACACCACCACCTCCTC<br>R-ATGCTCCGATCGCAAAAG	1.03
Purine nucleoside permease (*PUP3*)	F-GCCCACGGAAACAGTGAC<br>R-TCGCAAGCCACAACTGAA	0.99
Ubiquitin (Ub)	F-CATCAACTCCAACGGAAGCA<br>R-TCGGTCGGTCTTGTAAACGT	1.01
Iron transport multicopper oxidase	F-GTTTTTGGGACCGGCTTT<br>R-GCCGCTTTTGAGGGAAAT	0.99
Adenosine deaminase	F-TGAGTGCCACGACGAAAA<br>R-ACTCCACCATTGCCTCCA	1.01
